# Chest Radiograph Findings and Time to Culture Conversion in Patients with Multidrug-Resistant Tuberculosis and HIV in Tugela Ferry, South Africa

**DOI:** 10.1371/journal.pone.0073975

**Published:** 2013-09-06

**Authors:** James C. M. Brust, Andrew R. Berman, Benjamin Zalta, Linda B. Haramati, Yuming Ning, Moonseong Heo, Theo L. van der Merwe, Sheila Bamber, Anthony P. Moll, Gerald H. Friedland, N. Sarita Shah, Neel R. Gandhi

**Affiliations:** 1 Albert Einstein College of Medicine & Montefiore Medical Center, Bronx, New York, United States of America; 2 New Jersey Medical School, Newark, New Jersey, United States of America; 3 *Philanjalo* and Church of Scotland Hospital, Tugela Ferry, South Africa; 4 Centre for the AIDS Programme of Research in South Africa (CAPRISA), Durban, South Africa; 5 Yale University School of Medicine, New Haven, Connecticut, United States of America; 6 Emory University Rollins School of Public Health, Atlanta, Georgia, United States of America; National Institute of Allergy and Infectious Diseases, United States of America

## Abstract

**Background:**

The majority of patients with multidrug-resistant tuberculosis (MDR-TB) in South Africa are co-infected with HIV, but the radiographic features of MDR-TB and their relationship with time to sputum culture conversion in the antiretroviral therapy era have not been described.

**Methods:**

We reviewed baseline chest radiographs for 56 patients with MDR-TB from a rural area of South Africa. We analyzed the association of cavities, consolidation, pleural effusion and hilar lymphadenopathy with time to sputum culture conversion, adjusting for HIV status, baseline sputum smear and CD4 count.

**Results:**

Of the 56 subjects, 49 (88%) were HIV-positive, with a median CD4 count of 136 cells/mm^3^ (IQR 65-249). Thirty-two (57%) patients were sputum smear positive. Twenty-two (39%) patients had a cavity and 37 (66%) patients had consolidations. Cavitary disease and consolidations were each associated with longer time to culture conversion on bivariate analysis but not after adjusting for sputum smear status (aORs 1.79 [0.94-3.42] and 1.09 [0.67-1.78], respectively). Positive baseline sputum smear remained independently associated with longer time to conversion (aOR 3.45 [1.39-8.59]). We found no association between pleural effusion or hilar lymphadenopathy and time to conversion. Seventy-nine percent of patients were cured at the end of treatment.

**Conclusions:**

Despite high rates of HIV co-infection and advanced immunodeficiency, the majority of patients had severe pathology on baseline chest radiograph. Nevertheless, culture conversion rates were high and treatment outcomes were favorable. Cavitation and consolidation do not appear to have an independent association with time to culture conversion beyond that of baseline sputum smear status.

## Introduction

Multidrug-resistant tuberculosis (MDR-TB), defined as resistance to at least isoniazid and rifampin, remains a growing threat to TB control worldwide and is associated with high mortality—especially in the setting of HIV co-infection [1,2]. Treatment for MDR-TB is more expensive, more toxic and less effective than treatment for drug-susceptible TB, and must be given for approximately two years. Given the significant human and financial resources required for proper management of MDR-TB, several studies have attempted to identify clinical or biological risk factors for poor outcome, so that practitioners might stratify patients at the start of treatment and tailor their therapy accordingly [3,4]. Chest radiography, which is rapid and inexpensive, could potentially identify those with severe disease at the time of treatment initiation.

Even in resource-limited settings, most countries providing MDR-TB treatment have access to chest radiography, but the correlation of radiographic findings with clinical disease severity and its utility in predicting TB treatment outcomes remains unclear. Although previous studies have examined the relationship between chest radiograph findings and response to TB treatment, drug-resistance and HIV co-infection may complicate this relationship. Decreased time to sputum culture conversion has been an useful early predictor of successful final treatment outcome and several studies have found that cavitary disease on chest radiograph is associated with a longer time to culture conversion in drug-susceptible TB [5]. Notably, however, these and other studies have also shown that while patients with HIV are less likely to have cavitary disease, they are more likely to have poor outcomes from TB [6–8]. In addition, most studies examining predictors of sputum culture conversion were done in patients with drug-susceptible TB [9–11].

Among the few studies in MDR-TB, Holtz et al, found that cavitary disease on baseline chest radiograph was associated with a longer time to sputum culture conversion [12], but this study had only one patient (<1%) who was HIV-positive [13]. Whether baseline radiographic findings predict time to sputum culture conversion in patients with MDR-TB and HIV, therefore, remains unknown. The objective of the current study was to examine this relationship in a group of patients with MDR-TB in a rural area of South Africa with very high HIV seroprevalence.

## Methods

### Setting

Tugela Ferry is a resource-limited, rural area in KwaZulu-Natal (KZN) province, South Africa with a population of approximately 160,000 Zulu people. The case notification rate of TB is over 1,100 per 100,000 population and MDR-TB incidence was 118 per 100,000 in 2007 [2]. HIV prevalence among women seeking antenatal care is estimated at 37%. Patients with suspected or confirmed MDR-TB are referred to a regional, decentralized specialty center for further evaluation and treatment [14]. Since 2005, routine mycobacterial culture and drug-susceptibility testing (DST) has been available for all TB suspects in Tugela Ferry. This differs from other district hospitals in South Africa, and from that recommended by national policy, which reserves culture and DST for patients who are either being re-treated for TB, or who are failing first-line therapy [15].

Patients were treated with a standardized regimen of kanamycin, ofloxacin, ethionamide, ethambutol, pyrazinamide, and cycloserine (intensive phase) for 4 months following culture conversion (defined as two consecutive negative cultures at least one month apart) and a minimum of 6 months. This regimen was continued, without kanamycin, for an additional 18 months (continuation phase). Medications were dosed by weight and modified in response to severe adverse effects. Third-line TB drugs and surgical treatment were not used. Patients with resistance to either a fluoroquinolone or one of the second-line injectable medications (kanamycin, amikacin, or capreomycin) were transferred to the TB referral hospital in Durban and were not eligible for this study.

Most patients were hospitalized briefly to initiate MDR-TB therapy and were then discharged to continue treatment on an ambulatory basis. Patients were seen monthly at the decentralized MDR-TB clinic and sputum cultures were performed monthly throughout the course of treatment.

Sputum smears were examined with auramine fluorescent microscopy. After decontamination, sputum was inoculated in one mycobacteria growth indicator tube (MGIT) broth and on one Middlebrook 7H10 agar plate. The broths were incubated at 37°C in an automated incubator. Agar plates were sealed in CO_2_-permeable plastic bags and incubated in 5% CO_2_ at 37°C. Acid-fast microscopy was done on each positive MGIT broth. Those containing acid-fast bacilli were subcultured on Middlebrook 7H10 agar. Primary Middlebrook agar plates were read weekly for 3 weeks or until growth was observed. Microscopy was done to confirm the presence of acid-fast bacilli and all positive cultures were identified as *M. tuberculosis* by means of niacin and nitrate reductase tests.

Beginning in 2008, the hospital in Tugela Ferry began routinely scanning chest radiographs for all patients with TB. Using an EPSON 10000XL photographic and document scanner with transparency attachment, radiographs were scanned at a minimum of 350 dpi, in 16 bit black and white, using SilverFast EpsonIT8 software (Vers. 6.6.0r5) by Laser Soft Imaging.

### Population

Patients were eligible for inclusion in the study if they (1) had culture-confirmed pulmonary MDR-TB, (2) initiated MDR-TB therapy at the regional referral center and (3) had a scanned copy of their baseline posterior-anterior or anterior–posterior chest radiograph, taken prior to the initiation of MDR-TB therapy. When patients had multiple scanned radiographs on file, we selected the one performed as close as possible to treatment initiation, up to a maximum of 90 days prior.

### Chest radiograph analysis

Radiographs were interpreted by a single chest radiologist (B.Z.) who was blinded to the subjects’ clinical/demographic characteristics and treatment outcome. A standardized case report form was used to document the presence of cavities, consolidation, nodules, hilar lymph node enlargement, mediastinal lymph node enlargement, pleural effusion, pleural calcification, volume loss, and miliary disease. The location of any cavities or consolidation was specified by dividing the lungs into six zones (upper/middle/lower for each lung). If hilar lymph nodes were enlarged, the presence of unilateral or bilateral enlargement was noted. As a quality check, the primary radiologist read each film a second time, two weeks after the initial read, blinding himself to his original interpretation. All of these reads were concordant with the earlier interpretation. Any questionable findings were reviewed by a second radiologist (L.B.H.).

### Radiograph scoring

A cavity score and a consolidation score were each generated as follows: 0 = no cavity/consolidation present; 1=cavity/consolidation present in one lung zone; 2=cavity/consolidation present in at least two lung zones (unilateral or bilateral). Similarly, we created a score for hilar lymphadenopathy wherein 0 = no lymphadenopathy; 1 = unilateral; 2 = bilateral.

### Outcome scoring

Many patients suspected of having MDR-TB are placed on first-line TB treatment while awaiting the results of sputum culture and drug-susceptibility testing. As a result, although the initial culture may demonstrate MDR-TB, some of these patients have a negative repeat culture by the time they initiate MDR-TB treatment (possibly due to activity of ethambutol and/or pyrazinamide). We hypothesized that patients who convert their culture in this manner have less severe disease than those who are still culture-positive when they start MDR-TB treatment. We therefore created an ordinal culture conversion score in which 0 = conversion before MDR-TB treatment initiation; 1 = conversion within 60 days of initiating MDR-TB treatment; 2 = conversion after 60 days of initiating MDR-TB treatment, or no conversion at all. Time to conversion was defined as the number of days from initiation of MDR-TB therapy to the first of two negative cultures taken at least one month apart. The cut-point of 60 days was chosen because this was the median time to culture conversion seen both in our prior study of MDR-TB/HIV co-infection and in the HIV-negative literature [12,16].

### Statistical analysis

We used frequency analysis and chi-square testing to identify associations between clinical characteristics and radiographic findings. We examined the association of each of four radiographic findings (cavity score, consolidation score, effusion (yes/no) and hilar lymph node score) with the outcome of culture conversion score. We used a log-linear regression model assuming a multinomial distribution with a cumulative logit link function in which the primary predictor variable was the radiographic finding (e.g., cavity score) and the dependent variable was the culture conversion score. We adjusted each of the models for three clinical variables: HIV status, baseline sputum acid-fast bacilli (AFB) smear (positive/negative) and baseline CD4 count (≤50, 51-200, and >200 cells/mm^3^; at the time of MDR-TB treatment initiation).

The study was approved by the Institutional Review Boards at Albert Einstein College of Medicine, the University of KwaZulu-Natal, and Yale University, and by the KwaZulu-Natal Department of Health. The requirement for informed consent was waived by the IRBs listed above, as all patient data had been previously collected during the course of routine medical care and the study did not pose any additional risks to the patients.

## Results

**Figure 1:**
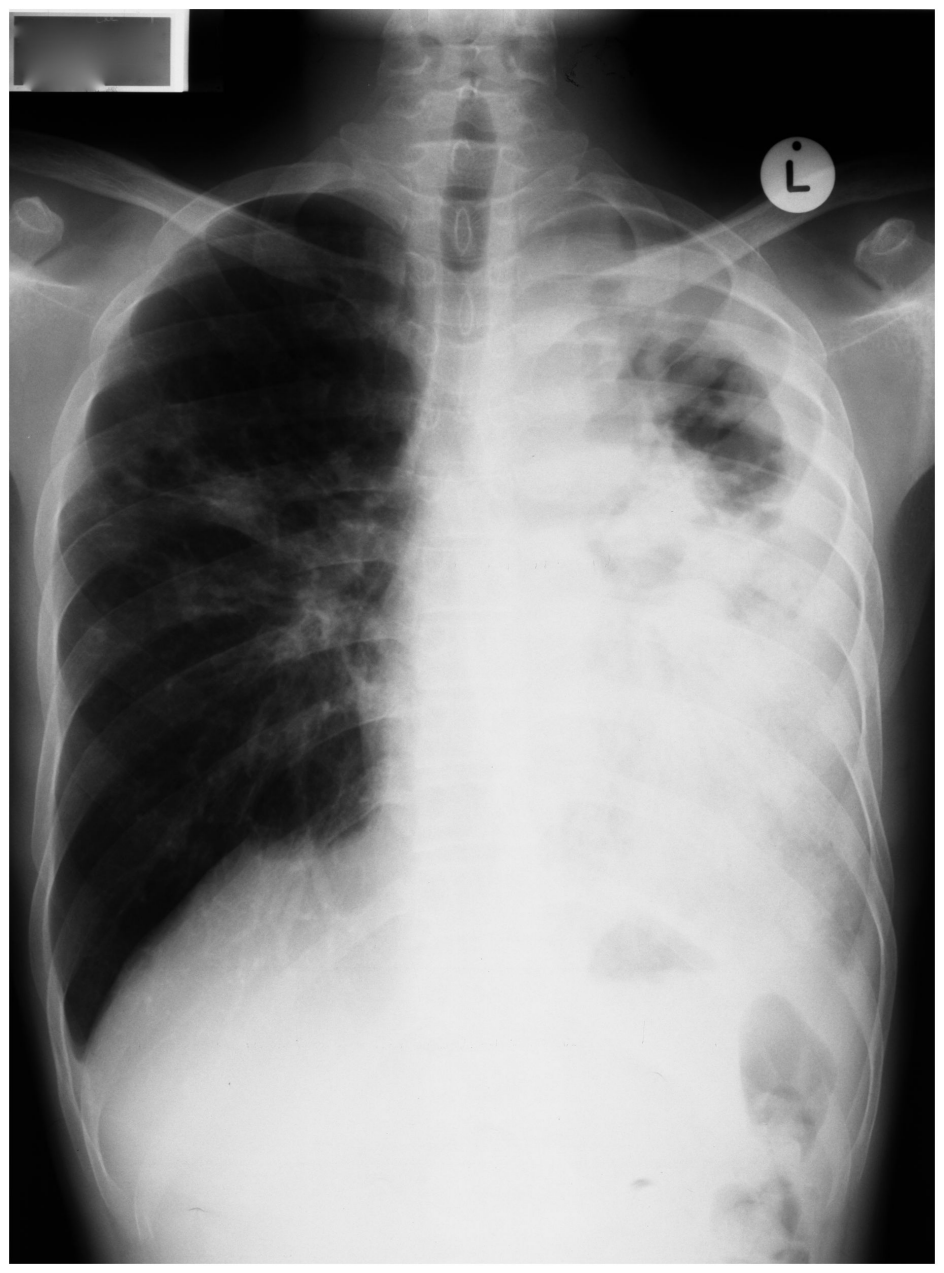
Chest radiograph showing left-sided pleural effusion, consolidation and both hilar and mediastinal lymphadenopathy.

Between February 2008 and May 2010, 97 patients from Tugela Ferry with culture-confirmed MDR-TB initiated therapy at the decentralized MDR-TB clinic. Of these, 56 (58%) had an available, scanned copy of their baseline chest radiograph (Figure 1). Patients with available radiographs did not differ from those without radiographs with respect to age, gender, HIV status, CD4 count, receipt of antiretroviral therapy (ART), or smear status (data not shown). Thirty-three (59%) patients were female and the median age was 36 (IQR 30-42) years. Forty-nine (88%) patients were HIV-positive with a median CD4 count of 136 cells/mm^3^ (IQR 65-249) at the time of MDR-TB treatment initiation. Of those who were HIV-positive, 40 (82%) were already receiving ART at the time of MDR-TB initiation and an additional 6 (12%) started ART during MDR-TB therapy. Thirty two (57%) patients were sputum AFB smear positive and 24 (43%) patients had been previously treated for TB (Table 1).

**Table 1:** Patient baseline characteristics (n=56).

**Characteristic**	**N (%)**
Female	33 (59)
Median Age, years (IQR)	36 (30-42)
Sputum Smear AFB-positive	32 (57)
Previous TB*	24 (43)
Previous MDR treatment	2 (4)
BMI^†^, median (IQR)	17.7 (16.1-21.6)
HIV-positive	49 (88)
Baseline CD4 available (% of HIV-positive)	42 (86)
Median baseline CD4, cells/ul (IQR)	136 (65-249)
On ART at time of MDR-TB treatment initiation (% of HIV-positive)	40 (82)
Days on ART at time of MDR-TB treatment initiation, median (IQR)	75 (3-181)
Started on ART during MDR-TB treatment (% of HIV-positive)	6 (12)

BMI: body mass index; IQR: interquartile range; ART: antiretroviral therapy

* Does not include first-line treatment for current TB episode prior to diagnosis of MDR-TB

† Baseline height and weight only available for 32 patients

### Radiographic findings

Radiographs were taken a median of 2 days (IQR 2-6) prior to initiating MDR-TB treatment and no radiographs preceded receipt of first-line TB treatment. Only one patient had a normal chest radiograph. Twenty-two (39%) patients had a cavity, of whom 6 (27%) had cavities in more than one lung zone (Table 2). Of those with cavities, 20 (91%) had an upper lobe cavity and 15 (68%) had cavities exclusively in the upper lobes. Thirty seven (66%) patients had pulmonary consolidations, of whom 23 (62%) had consolidations in more than one lung zone. Hilar lymphadenopathy was common, and of the 40 (71%) patients with enlarged hilar lymph nodes, 16 (40%) had bilateral enlargement. Nineteen (34%) patients had pleural effusions. Among patients with a CD4 count ≤50 cells/mm^3^, all had hilar lymphadenopathy and none had cavities. Additional radiographic findings are shown in Table 2.

### Sputum culture conversion

Thirteen (23%) patients had a negative sputum culture at the time of MDR-TB treatment initiation (Table 3). Eighteen (32%) patients converted their culture within 60 days and 25 (45%) either converted their culture after 60 days or not at all. In bivariate analysis of each radiographic feature with culture conversion score (0, 1, or 2), cavity score was associated with longer time to culture conversion (OR 2.17; CI 1.29-3.64), as was consolidation score (OR 1.60; CI 1.11-2.32) (Table 4). We found no association, however, between presence of pleural effusion (OR 1.12; CI 0.59-2.11) or hilar lymph node enlargement (OR 1.35; CI 0.91-2.02) and time to culture conversion. Positive sputum smear was associated with longer time to conversion (OR 3.93; CI 1.92-8.07) but neither HIV status nor CD4 count were associated with time to conversion. In addition, while positive sputum smear was strongly associated with cavity score (Fisher’s Exact Test p=0.005), we found no association between positive sputum smear and either HIV status (p=0.69) or CD4 count (p=1.0).

**Table 2:** Characteristics of baseline chest radiographs.

**CXR Feature**	**All subjects (n=56) N (%)**	**HIV-positive (n=49) N (%)**	**HIV-negative (n=7) N (%)**	p-value^*^
Cavity	22 (39)	16 (33)	6 (86)	0.01
One lung zone, (% of those with cavity)	16 (73)	11 (69)	5 (83)	
Two or more lung zones (unilateral or bilateral)	6 (27)	5 (31)	1 (17)	
Consolidation	37 (66)	32 (65)	5 (71)	1.0
One lung zone, (% of those with consolidation)	14 (38)	13 (41)	1 (20)	
Two or more lung zones (unilateral or bilateral)	23 (62)	19 (59)	4 (80)	
Pleural Effusion	19 (34)	18 (37)	1 (14)	0.40
Hilar lymph nodes	40 (71)	36 (73)	4 (57)	0.39
Unilateral	24 (60)	21 (58)	3 (75)	
Bilateral	16 (40)	15 (42)	1 (25)	
Calcified Lymph nodes	22 (39)	19 (39)	3 (43)	1.0
Volume loss/collapse/bronchiectasis	32 (57)	27 (55)	5 (71)	0.68
Pleural calcification	6 (11)	6 (12)	0 (0)	1.0
Miliary Disease	2 (4)	2 (4)	0 (0)	1.0
Mediastinal lymph nodes	41 (73)	36 (73)	5 (71)	1.0

* 2-tailed Fisher’s exact test.

After adjusting for baseline sputum smear, HIV status, and CD4 count, neither cavity nor consolidation score were significantly associated with longer time to conversion although there remained weak evidence of an association between cavity score and culture conversion. In this multivariate model, positive baseline sputum smear remained strongly and independently associated with later culture conversion, but we found no such association for HIV or CD4 count.

**Table 3:** Culture conversion and treatment outcomes.

**CultureConversion**	**N (%)**
Converted sputum culture to negative	53 (95)
Converted culture prior to initiation of second-line regimen	13 (23)
Converted while receiving second-line regimen	40 (71)
Median days to culture conversion (IQR)*	66 (43-96)
**TreatmentOutcomes**	
Final Outcome reached	56 (100)
Cured	44 (79)
Died	4 (7)
Transferred Out	4 (7)
Defaulted	3 (5)
Treatment failure	1 (2)

IQR: interquartile range

* Measured from date of MDR-TB treatment initiation to the first of two negative sputum cultures taken at least one month apart.

### Treatment Outcomes

Final treatment outcomes were available for all 56 patients and of these, 44 (79%) were cured, four (7%) died, 3 (5%) defaulted, 1 (2%) failed therapy and 4 (7%) were transferred to the provincial TB specialty hospital in Durban (Table 3). We performed bivariate logistic regression to identify predictors of treatment success (defined as cure or treatment completion). We found no association between the radiographic findings, HIV status, smear status or CD4 count and final treatment outcome.

## Discussion

This study, which examined the relationship between baseline chest radiographic findings and time to sputum culture conversion in MDR-TB, has several important findings. Despite extensive pathology on baseline chest radiograph, and advanced immunodeficiency in the majority of patients, MDR-TB treatment outcomes were favorable in this cohort. All but three patients (5%) achieved culture conversion and 79% of patients were cured at the end of treatment. Median time to culture conversion was 66 days, which is similar to our earlier published findings as well as reports in the HIV-negative MDR-TB literature [12,16].

**Table 4:** Association of radiographic findings and clinical characteristics with culture conversion score^*^ using log-linear regression.

**Characteristic**	**Crude Odds Ratio**	**95% Confidence Interval**	**Adjusted Odds Ratio**	**95% Confidence Interval**
Cavity Score^†^	**2.17**	**1.29-3.64**	1.79	0.94-3.42
Consolidation Score^‡^	**1.60**	**1.11-2.32**	1.09	0.67-1.78
Pleural effusion	1.12	0.59-2.11	−	−
Hilar lymphadenopathy	1.35	0.91-2.02	−	−
HIV	0.83	0.32-2.15	1.83	0.60-5.58
Baseline sputum smear	**3.93**	**1.92-8.07**	**3.45**	**1.39-8.59**
CD4 group^§^	1.16	0.74-1.82	1.21	0.71-2.08

* Culture conversion score: 0 = conversion prior to initiation of MDR-TB treatment; 1 = conversion within 60 days of initiating MDR-TB treatment; 2=conversion >60 days after initiating MDR-TB treatment OR lack of culture conversion.

† Cavity score: 0 = no cavity; 1 = cavity in one lung zone; 2 = cavity in >1 lung zone.

‡ Consolidation score: 0 = no consolidation; 1 = consolidation in one lung zone; 2 = consolidations in >1 lung zone.

§ CD4 groups: ≤50, 51-200, and >200 cells/mm^3^; HIV-negative subjects were included in the highest CD4 group.

**Bold**: p<0.05

Although the presence and degree of cavitary disease on baseline chest radiograph was associated with a longer time to culture conversion in MDR-TB in bivariate analysis, this effect did not retain statistical significance after adjusting for baseline sputum smear status. In the multivariate model, smear status was the only independent predictor of culture conversion, suggesting that smear-positivity—which reflects bacillary burden in the lung—may mediate the effect of cavitation on time to culture conversion in our analysis. Other studies have examined the prognostic significance of cavitary disease and have found an inverse relationship with likelihood of culture conversion [5,17]. To our knowledge, however, this is the largest radiographic study of MDR-TB/HIV co-infection in the ART era.

Several studies have shown that radiographic findings from TB differ between patients with and without HIV [7,8,18–20]. Perlman et al. found that severity of immunosuppression from HIV was inversely associated with the presence of cavities on chest radiograph in patients with drug-susceptible TB [21]. Only 10% of patients in their cohort had cavitary disease, but those with CD4 <200 cells/mm^3^ were significantly less likely to have cavities and more likely to have hilar/mediastinal lymphadenopathy. We found a similar relationship between CD4 count and radiographic findings; all of the patients with a CD4 count ≤50 cells/mm^3^ had hilar lymphadenopathy and none had cavities. Overall, however, a greater proportion of our patients (39%) had cavitary disease compared to those reported by Perlman. Angthong et al. also found a higher prevalence of cavitary disease among Japanese HIV-infected patients with a CD4 count >200 cells/mm^3^ and drug-susceptible TB, but their study population only included 5 patients with a high CD4 count [22]. Fishman et al., by contrast, found no difference in the presence of cavities between patients with a CD4 count greater than, versus less than 100 cells/mm^3^ [23]. We found no association between CD4 count and location of cavity, but our analysis was limited by small numbers within the subgroups.

In their study from New York in the mid-1990’s, Telzak et al. found that cavitary disease and baseline smear, but not HIV, were associated with a prolonged time to culture conversion [17]. This cohort was a mix of drug-susceptible and MDR-TB cases, but similar to our population, most had no history of prior TB treatment. Investigators in Spain found that HIV infection was associated with less frequent cavitary disease and a shorter time to sputum smear conversion, but the authors did not report CD4 counts [11]. They, too, found that the presence of cavities on chest radiograph was associated with a prolonged time to sputum smear conversion, consistent with our findings. We suspect that the unifying mechanism relating HIV infection, cavitary disease and sputum culture conversion is the lung bacillary burden, as indicated by the sputum smear. HIV co-infected patients with higher CD4 counts are more likely to develop cavitary disease and are thus more likely to have a high bacillary burden [24] and to be sputum smear-positive at baseline [25]. This, in turn, prolongs the time to culture conversion when receiving therapy but does not necessarily predict poor final treatment outcomes.

Our findings must be taken in the context of the local drug-resistant tuberculosis epidemic. In studies from several countries, the MDR-TB subjects had received multiple courses of TB, and even MDR-TB therapy [12,26–28]. Our population, by contrast, was not heavily treatment-experienced. We have previously shown that the epidemic in Tugela Ferry has been driven primarily by transmission—often in the hospital setting [29]—and consistent with these earlier findings, the majority of subjects in this study had never been previously treated for TB. In light of this evidence suggesting recent, primary MDR-TB infection, high rates of HIV co-infection and low CD4 counts, the extensive pathology seen on chest radiographs in this cohort is surprising.

This study has several limitations. First, we did not have radiographs from a control population of drug-susceptible TB patients for comparison. Nevertheless, when compared to other studies in patients with drug-susceptible TB or MDR-TB but without HIV co-infection, our findings shed further light on the interplay between TB disease, drug-resistance, HIV infection and radiographic findings. Second, our sample size was small, and the borderline significance of the association between cavitary disease and time to conversion may reflect a lack of power. With a larger sample size, this association—which was significant on bivariate analysis—may have retained significance in the adjusted model. Similarly, the number of HIV-negative subjects in our cohort was also very small, limiting our ability to examine independent effects of HIV-infection. A third limitation is that follow-up radiographs were not available for most patients, and as a result, we were unable to examine how changes in radiographic findings during treatment might correlate with, or predict clinical course and culture conversion. Fourth, because a majority of patients initiated ART several weeks prior to starting MDR-TB therapy, we were unable to exclude the possibility that some radiographic findings represented immune reconstitution inflammatory syndrome (IRIS). Finally, post-treatment surveillance results are not available, and it is possible that patients with extensive cavitary disease and longer time to culture conversion may be at higher risk of relapse after “cure.” Future studies are needed to answer this question and to determine if such patients should receive a prolonged intensive phase, prolonged continuation phase, or other modification in treatment.

The results of our study suggest that the presence of cavities on baseline chest radiograph is associated with prolonged time to sputum culture conversion. This relationship is likely mediated by a high bacillary burden in the lung and positive sputum smear for AFB. The presence and degree of lymphadenopathy and pleural effusions in this cohort were not associated with time to culture conversion. Chest radiographs remain a useful clinical tool for monitoring response to therapy and diagnosing the immune reconstitution inflammatory syndrome, and a baseline radiograph should be obtained in all patients for later comparison. As a predictive tool in this South African population, however, radiographs may not have an independent association with time to sputum culture conversion beyond that of baseline sputum smear status.
